# Antioxidant Activity in Two Pearl Millet (*Pennisetum typhoideum*) Cultivars as Influenced by Processing

**DOI:** 10.3390/antiox3010055

**Published:** 2014-02-12

**Authors:** Florence Suma Pushparaj, Asna Urooj

**Affiliations:** Department of Studies in Food Science and Nutrition, University of Mysore, Mysore 570006, India; E-Mail: asna321@sify.com

**Keywords:** pearl millet, Kalukombu, Maharashtra Rabi Bajra, antioxidant activity, flavonoids, phytic acids, tannin, processing

## Abstract

Research on the effect of processing on the retention of bioactive components with potential antioxidant activity is gaining importance. The objective of this investigation was to evaluate the effect of various processing methods (milling, boiling, pressure cooking, roasting and germination respectively) on the antioxidant components as well as the antioxidant activities in the commonly used pearl millet cultivars—Kalukombu (K) and Maharashtra Rabi Bajra (MRB). The methanolic extracts of processed pearl millet flours were analyzed for 1,1-diphenyl-2-picrylhydrazyl (DPPH) free radical scavenging activity, reducing power assay (RPA) and ferric reducing antioxidant power (FRAP) assays respectively. The samples were also evaluated for tannin, phytic acid and flavonoid content which was then correlated with the antioxidant activity assayed using three methods. The results indicated that the bran rich fraction showed high antioxidant activity (RPA) owing to high tannin, phytic acid and flavonoid levels. Heat treatments exhibited significantly (*P* ≤ 0.05) higher antioxidant activity (DPPH scavenging activity and RPA) reflecting the high flavonoid content. Processing did not have any significant effect on the FRAP activity of pearl millet. The data on the correlation coefficient suggests that DPPH radical scavenging activity and reducing power assay in the K variety was largely due to the presence of flavonoid content, however in MRB, no relationship was found between antioxidant activities and antioxidant components.

## 1. Introduction

The term “phytochemicals” or “plant chemicals” refers to every naturally occurring chemical substance present in plants, which also has a potential for antioxidant activity. Antioxidants play an important role in the body’s defense system against reactive oxygen species (ROS), which are the harmful byproducts generated during normal cell aerobic respiration [1]. In foods, antioxidants prevent undesirable changes in flavor and nutritional quality of a product [2]. Several methods have been developed to measure “antioxidant activity”. Commonly used assays are reducing power assay (RPA), ferric reducing antioxidant power (FRAP) and 1,1-diphenyl-2-picrylhydrazyl (DPPH) free radical scavenging activity. There are two basic categories of antioxidants, namely, natural and synthetic. Natural antioxidants are phenolic compounds (flavonoids, phenolic acids), nitrogen compounds (alkaloids, chlorophyll derivatives, amino acids, and amines), carotenoids and ascorbic acid. Butylated hydroxyanisole (BHA) and butylated hydroxytoluene (BHT) are commonly used synthetic antioxidants that have been in use since the beginning of this century. Restrictions on the use of these compounds, however, are being imposed because of their carcinogenicity [3,4]. Thus, natural antioxidants have gained considerable interest in recent years.

Cereals and millets are the most commonly consumed food items in India. They contain a wide range of phenolics which are good sources of natural antioxidants. Studies report that methanolic extracts from red sorghum showed higher antioxidant activity and contain higher polyphenolic levels compared to rice, foxtail millet, prosomillet and barley [5]. Bran, a byproduct of milling has antioxidant potential due to phenolic acids such as *p*-coumaric acid and vanillic acids that are concentrated in the bran portion of cereal kernels. Antioxidant activity of five bran extracts exhibited appreciable levels of total phenolics, flavonoids and DPPH radical scavenging activities [6]. Processing, such as soaking and roasting, have been shown to influence total phenolic, flavonoid and antioxidant contents in selected dry beans. Raw kodo millet and finger millet have higher DPPH radical scavenging activities. However, cooking of these millets by roasting or boiling reduced their antioxidant activity [7].

Millets contain phytic acid, tannins, and phenols which can contribute to antioxidant activity, important in health, ageing and metabolic diseases. Pearl millet (*Pennisetum typhoideum*) is the most widely grown type of millet. Nutritionally, pearl millet is superior to major cereals with reference to energy value, high quality proteins, fat and minerals such as calcium, iron, zinc. Besides, it is also a rich source of dietary fiber and micro nutrients [8,9]. While, extensive information is available on proximate composition and mineral accessibility, information on antioxidant activity and its influence on processing in pearl millet is scanty. Research on the effect of processing on retention of bioactive components with potential antioxidant activity is very important. The objective of this investigation was to evaluate the effect of various processing methods (milling, heat treatments and germination respectively) on antioxidant components as well as antioxidant activities of pearl millet extracts. The methanolic extracts of raw and processed pearl millet were analyzed for DPPH free radical scavenging activity; reducing power assay (RPA) and ferric reducing antioxidant power (FRAP) assays respectively. The samples were also evaluated for tannin, phytic acid and flavonoid content and were correlated with the antioxidant activity assayed using three methods.

## 2. Experimental Section

### 2.1. Materials

Certified varieties of Pearl millet—“Kalukombu” (K) and Maharashtra Rabi Bajra (MRB) were used for the study. “Kalukombu” (K) is a native variety traditionally grown by farmers in India (Karnataka, Tamilnadu and Maharashtra). This variety has not been improved by the modern plant breeding system. It is considered nutritionally very superior by the local people and is used as a food crop to make roti, dumpling and chapattis. The seeds of Kalukombu are small and elongated with persisting glumes/husk. “Maharashtra Rabi Bajra” (MRB) is a commercially grown hybrid developed by the modern improved plant breeding technique by a commercial seed company. It is basically a winter crop. The seeds are grey/slate colored, bold and round shaped without persisting glumes/husk [10].

Chemicals such as DPPH (1,1-diphenyl-2-picrylhydrazyl) were procured from HiMedia Lab Pvt. Ltd. (Mumbai, India). All other reagents used were of analytical grade.

### 2.2. Processing of Pearl Millet

*Milling*: The cleaned grains were pulverized using a plate mill to obtain whole flour (WF). A part of the whole flour was further sieved through a 44 mesh sieve (BSS). The “+” fraction was termed as the bran rich fraction (BRF) and the “–” fraction was termed as semi-refined flour (SRF).

Heat treatment: 

*Boiling*: Pearl millet grains were held in a pan of boiling water (1:1 grain to water, weight to volume basis) for 30 min.

*Pressure cooking*: Pearl millet grains were pressure cooked in water (1:1 grain to water, weight to volume basis) for 10 min (9.8 × 104 Pa).

*Roasting*: Pearl millet grains were roasted in an open pan for 10–15 min at 200 °C.

*Germination*: Pearl millet was soaked in water overnight. The water was drained and the grains were tied in a moist muslin cloth and left to sprout at room temperature for 72 h [11].

Each treatment was replicated twice. The wet heat treated and germinated grains were laid out on steel trays in thin layers of less than 2 cm. The trays were placed in a hot air oven and dried at 50 °C for 24 h. The samples were milled to flour using a hammer mill and stored in air tight polythene bags in a cool and dry place until use.

### 2.3. Preparation of Pearl Millet Extracts

Raw and processed pearl millet flours (15 g) were extracted with 200 mL of methanol in a mechanical shaker for 24 h, filtered and evaporated to dryness under reduced pressure in a rotary evaporator. The concentrated extract was re-dissolved with methanol to a concentration of 10 mg/mL and stored in the refrigerator until analysis. All the analyses were carried out in triplicates.

### 2.4. DPPH Radical Scavenging Activity

The ability of the methanolic extracts to scavenge free radicals was determined against a very stable free radical DPPH (1,1-diphenyl-2-picrylhydrazyl) determined spectrometrically [12] (Blois 1958). Aliquots of the sample extract at different concentrations were added to 1 mM methanolic solutions of DPPH. Each mixture was vortexed vigorously and left for 30 min at room temperature in the dark. The absorbance was measured at 517 nm and activity was expressed as percentage DPPH scavenging relative to control using the following equation:

DPPH scavenging activity (%) = (Absorbance of control − Absorbance of sample)/Absorbance of control × 100
(1)

### 2.5. Reducing Power Assay

The ability of methanolic extracts to reduce iron (III) to iron (II) was assessed by the method of Yildrim *et al.* [13]. The dried extract (125–1000 µg) in 1 mL of the corresponding solvent was mixed with 2.5 mL of phosphate buffer (0.2 M, pH 6.6) and 2.5 mL of potassium ferricyanide (K_3_Fe(CN)_6_; 10 g/L), and then the mixture was incubated at 50 °C for 30 min. After incubation, 2.5 mL of TCA (100 g/L) was added and the mixture was centrifuged at 1650 rpm for 10 min. Finally, 2.5 mL of the supernatant solution were mixed with 2.5 mL of distilled water and 0.5 mL of FeCl_3_ (1 g/L) and the absorbance was measured at 700 nm. High absorbance indicates high reducing power.

### 2.6. FRAP (Ferric Reducing Antioxidant Power Assay)

The FRAP assay was carried out according to the procedure of Benzine and Strai [14]. The FRAP reagent was prepared by mixing acetate buffer (25 mL, 300 mmol/L, pH 3.6), 10 mmol/L TPTZ solution (2.5 mL) in 40 mmol/L HCl and 20 mmol/L FeCl_3_ solution (2.5 mL) in proportions of 10:1:1 (v/v), respectively. The FRAP reagent was prepared fresh and warmed to 37 °C in a water bath prior to use. One hundred and fifty micro liters of the sample was added to the FRAP reagent (4.5 mL). The absorbance of the reaction mixture was than recorded at 593 nm after 4 min, the assay was carried out in triplicate. The standard curve was constructed using FeSO_4_ solution (0.5–10 mg/mL). The results were expressed as µmol Fe(II)/g dry weight of plant material.

### 2.7. Antioxidant Components

*Tannins*: Tannin content in the samples was measured using the method described by AOAC [15].

*Phytic acid* was extracted and determined according to the Thompson & Erdman method [16].

*Flavonoids*: The total flavonoid content of the samples was determined according to Miliauskass *et al.* [17] using rutin as a reference compound. One mL of plant extract in methanol (10 g/L) was mixed with 1 mL of aluminum trichloride in ethanol (20 g/L) and diluted with ethanol to 25 mL. The mixture was incubated at 20 °C and the absorbance was measured at 415 nm. The blank was prepared by diluting 1 mL plant extract and 1 drop acetic acid to 25 mL with ethanol. The flavonoid content in the samples was calculated using the following formula:

*X* = (*A* × *m*_o_ × 10)/(*A*_o_ × *m*) (2)

where: *X*—Flavonoid content, mg/g plant extract in Rutin Equivalents; *A*—Absorption of Sample; *A*_o_—Absorption of standard rutin solution; *m*—Weight of Sample; *m*_o_—Weight of rutin in the solution.

### 2.8. Statistical Analysis

The data was subjected to analysis of variance (ANOVA) test using SPSS 17.0 software (SPSS Inc., Chicago, IL, USA) and the differences between the means were compared for their significance. Correlation coefficients between antioxidant components and antioxidant activity were determined.

## 3. Results and Discussion

### 3.1. Yield of Methanolic Extracts (g/100 g)

Antioxidants are difficult to extract due to differences in the active compounds. A wide variation is seen in detecting antioxidant components due to differences in the polarity of the extracting solvents. Methanol is a relatively polar organic solvent and appears to be efficient in extracting compounds such as phenolics, flavonoids, and other polar material from millets [18,19]. Methanol was therefore selected as an extracting solvent in the present investigation. Table 1 exhibits the yield of methanolic extracts obtained from the raw and processed two pearl millet varieties. The K variety (5.8 g/100 g) had higher yield than MRB (4.4 g/100 g). Similar yields were reported for wheat and barley [2]. A wide variation in the yield as a result of processing was seen. Lower yields were seen in the millet subjected to heat treatments (boiling, pressure cooking, roasting) as well as in the bran rich fraction, while germination facilitated maximum extraction in both varieties; however this increase was not statistically significant.

**Table 1:** Yield of methanolic extracts obtained from processed pearl millet flours.

Processing	K	MRB
WF (Raw)	5.8 ^cd^ ± 1.20	4.4 ^bc^ ± 0.87
SRF	4.8 ^bc^ ± 0.30	3.7 ^abc^ ± 0.26
BRF	2.1 ^a^ ± 0.05	3.2 ^ab^ ± 0.15
B	2.4 ^ab^ ± 0.38	2.2 ^ab^ ± 0.14
PC	2.2 ^a^ ± 0.10	1.8 ^a^ ± 0.04
R	3.0 ^ab^ ± 0.03	2.3 ^ab^ ± 0.04
G	7.7 ^d^ ± 0.12	6.1 ^c^ ± 0.34

Values are mean ± SD (*n* = 4), Means with different superscripts (a, b, c, d) along the column are significantly different (*P* ≤ 0.05), MRB—Maharashtra Rabi Bajra. Values are mean ± SD (*n* = 4), WF—Whole flour, SRF—Semi refined flour, BRF—Bran rich fraction, B—boiling, PC—Pressure cooking, R—roasting, G—germination.

### 3.2. Antioxidant Components

Millets contain phytates, phenols, tannins, trypsin inhibitors and dietary fiber which act as “antinutrients” by chelating minerals. Tannins are naturally occurring polyphenolic compounds linked to reduce protein digestibility by forming complexes with proteins and inhibiting enzymes [20]. It has now been established that phytates, phenols and tannins present in cereals are also good sources of natural antioxidants important in health, aging and metabolic diseases [21]. Phytic acid occurring in the grains acts as an antioxidant by the formation of chelates with pro-oxidant transition metals. Although, phytic acid is generally regarded as an antinutrient due to its mineral binding activity it is known to reduce the risk of colon and breast cancer in animals [22]. Table 2 exhibits the tannin, phytic acid and flavonoid content of raw and processed pearl millet. The tannin contents of K and MRB expressed as tannic acid equivalents were 23 and 21 g/100 g of dry flour. Semi refining reduced the level of tannins in the SRF thus increasing its level in BRF suggesting its localization in the outer hulls of the grain. Millet subjected to various heat treatments lead to considerable reduction in tannin levels only in the K variety. On the other hand, the increase in the tannin content of the germinated flour extract in both varieties was pronounced, however, statistically not significant for the MRB variety.

**Table 2:** Effect of processing on antioxidant components of pearl millet.

Processing treatments	Tannins (g/100 g) ^†^		Phytic acid (g/100 g)		Flavonoids (mg/g) ^£^	
	K	MRB	K	MRB	K	MRB
WF (Raw)	0.23 ^c^ ± 0.01	0.21 ^a^ ± 0.02	0.78 ^c^ ± 0.02	0.57 ^cd^ ± 0.07	0.27 ^ab^ ± 0.04	0.21 ^a^ ± 0.02
SRF	0.21 ^b^ ± 0.01	0.19 ^a^ ± 0.01	0.66 ^b^ ± 0.06	0.33 ^ab^ ± 0.09	0.20 ^a^ ± 0.07	0.18 ^a^ ± 0.02
BRF	0.31 ^d^ ± 0.01	0.32 ^b^ ± 0.01	0.99 ^d^ ± 0.06	0.61 ^d^ ± 0.10	0.19 ^a^ ± 0.01	0.18 ^a^ ± 0.00
Bo	0.18 ^a^ ± 0.01	0.19 ^a^ ± 0.01	0.57 ^b^ ± 0.11	0.39 ^ab^ ± 0.04	0.32 ^abc^ ± 0.1	0.13 ^a^ ± 0.00
PC	0.18 ^a^ ± 0.01	0.22 ^a^ ± 0.02	0.58 ^b^ ± 0.04	0.60 ^d^ ± 0.06	0.36 ^bc^ ± 0.06	0.13 ^a^ ± 0.01
Ro	0.20 ^b^ ± 0.00	0.23 ^a^ ± 0.03	0.43 ^a^ ± 0.06	0.45 ^bc^ ± 0.11	0.44 ^c^ ± 0.10	0.27 ^a^ ± 0.07
G	0.36 ^e^ ± 0.01	0.28 ^a^ ± 0.01	0.37 ^a^ ± 0.07	0.26 ^a^ ± 0.01	0.17 ^a^ ± 0.02	0.10 ^a^ ± 0.02

**^†^—**g tannic acid equivalents/100 of dry flour, £—mg rutin equivalents/g of extract, values are mean ± SD (*n* = 4), means with different superscripts (a, b, c, d) along the column are significantly different (*P* ≤ 0.05), K—Kalukombu, MRB—Maharashtra Rabi Bajra, WF—Whole flour, SRF—Semi refined flour, BRF—Bran rich fraction, Bo—boiling, PC—Pressure cooking, Ro—roasting, G—germination.

K exhibited higher phytic acid content (78 g/100 g) compared to MRB (57 g/100 g). Semi refining significantly reduced the corresponding values to 66 and 33 g/100 g for K and MRB respectively. The differences were mainly found concentrated in the bran rich fraction (99 and 61 g/100 g K and MRB respectively). Phytic acid content significantly (*P* ≤ 0.05) decreased due to heat treatments although the maximum reduction was seen in the germinated millet.

The flavonoid contents of raw and processed millet extracts were expressed as mg rutin equivalents per gram of the extract (10 mg/mL). The flavonoid content was highest in K (27 mg/g) followed by MRB (21 mg/g). The milling fractions (SRF and BRF) irrespective of varietal differences, showed a reduction in the flavonoid content, nonetheless, this decrease was not statistically significant. Unlike tannins and phytic acid, the flavonoid levels considerably increased due to heat treatments (boiling, pressure cooking, roasting) while, germination did not alter the flavonoid content of pearl millet. Our results are in agreement with the findings of Lachman *et al.* [23], that boiling and other heat treatment increased the total anthocyanin content of flesh-colored potatoes.

### 3.3. Radical Scavenging Activity by DPPH

**Figure 1:**
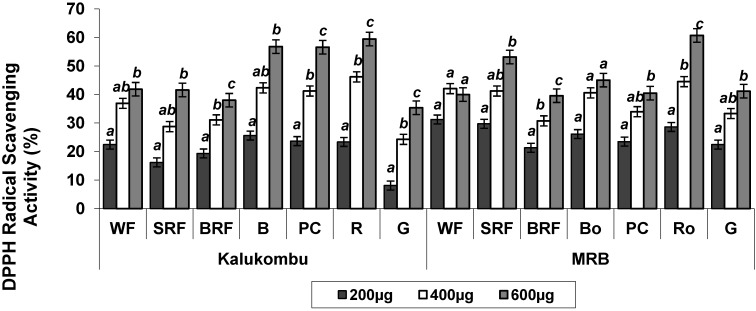
Effect of processing on the radical scavenging activity of pearl millet extracts by 1,1-diphenyl-2-picrylhydrazyl (DPPH) at various concentrations.

Figure 1 summarizes data on DPPH radical scavenging activity of raw and processed pearl millet. In the DPPH assay, the color stable DPPH radical is reduced in the presence of an antioxidant which donates hydrogen to non-radical DPPH–H [24]. DPPH free radical scavenging activity was studied at three concentrations (200 µg, 400 µg and 600 µg). Radical scavenging activity varied with the processing methods used and was concentration dependent. The greatest activity was obtained at a higher concentration of 600 µg in the raw and processed flour extracts. The antioxidant activity of K and MRB which was 22% and 31% at 200 µg considerably increased to 42% and 40% respectively at 600 µg. As affected by processing, the activity was in the order: G < WF < BRF < SRF < Bo < PC < Ro for K and WF < BRF < PC < G < Bo < SRF < Ro for MRB. Heat treatments such as boiling; roasting and pressure cooking exhibited significantly higher antioxidant activity as compared to the raw flour. Similar findings were reported for little millet where roasting of the millet enhanced its radical scavenging activity (95.5%), compared to germinated (91.7%) and steamed (93.4%) millet [25]. In contrast, cooked peppers showed a marked reduction in the radical scavenging activity when cooked for 30 min in boiling water. This may be due to leaching of antioxidant compound from the pepper into the cooking water during the prolonged exposure to water and heat [26]. It was noteworthy that significantly high radical scavenging activity in heat treated millet had the lowest yield, whilst, germinated millet extracts which showed the lowest activity had the highest yield.

Bars with different superscripts (a, b, c, d) are significantly different (*P* ≤ 0.05), MRB—Maharashtra Rabi Bajra, WF—whole flour, SRF—semi refined flour, BRF—Bran rich fraction, Bo—boiling, PC—pressure cooking, Ro—roasting, G—germination.

### 3.4. Reducing Power Assay

**Figure 2:**
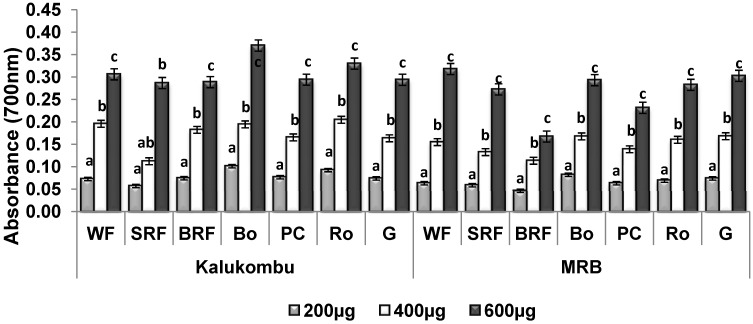
Effect of processing on the reducing power of pearl millet extracts at various concentrations

The ability of methanolic extracts to reduce iron (III) to iron (II) is shown in Figure 2. High absorbance indicated high reducing power. It was observed that the results followed a trend similar to DPPH free radical scavenging activity. A concentration dependent reducing power was found for the all processed millet. The reducing power of K and MRB flour extracts at 200 µg concentration were *A*_700_ = 0.07 and 0.06 respectively. The corresponding values increased to *A*_700_ = 0.31 and 0.32 respectively at 600 µg. The respective bran rich fraction, roasted and boiled millet extracts of the K variety exhibited an increase in absorbance (*A*_700_ = 0.34, 0.39 and 0.47, respectively) compared to the raw millet extract (*A*_700_ = 0.31). However, processing did not alter the antioxidant activity (reducing power) of MRB.

Different superscripts (a, b, c) on each bars are significantly (*P* ≤ 0.05) different; MRB—Maharashtra Rabi Bajra, WF—whole flour, SRF—semi refined flour, BRF—bran rich fraction, Bo—boiling, PC—pressure cooking, Ro—roasting, G—germination.

### 3.5. Ferric Reducing Antioxidant Power (FRAP)

FRAP assay is a novel method for assessing antioxidant power where the ferric reducing ability of sample extract is tested. Ferric to ferrous ion reduction at low pH causes a colored ferrous-tripyridyltriazine complex to form. FRAP values are obtained by comparing the absorbance change at 593 nm in test reaction mixtures with those containing ferrous ions in known concentration Benzine and Strai [14]. In this study, FRAP was calculated using the equation *Y* = 0.2453*x*, where *x* is the OD of the sample. The ferric reducing ability of the extracts was higher in K (2.24) followed by MRB (1.85) (Table 3). Pressure cooked K variety and germinated MRB exhibited lowest activity, but was not statistically significant. Overall, the processing methods employed in this study did not cause any significant changes in the activity (FRAP) of the millet.

**Table 3:** Effect of processing on FRAP of two pearl millet varieties.

Processing	Kalukombu	MRB
Whole flour (Raw)	2.24 ^ab^ ± 0.57	1.85 ^ab^ ± 0.21
SRF	2.89 ^b^ ± 0.54	1.63 ^a^ ± 0.20
BRF	2.08 ^ab^ ± 0.05	1.72 ^ab^ ± 0.09
Bo	2.18 ^ab^ ± 0.03	1.99 ^ab^ ± 0.07
PC	1.54 ^a^ ± 0.11	1.80 ^ab^ ± 0.00
Ro	2.58 ^b^ ± 0.33	2.17 ^b^ ± 0.12
G	2.69 ^b^ ± 0.07	1.66 ^a^ ± 0.23

Values are mean ± SD (*n* = 6), Means with different superscripts (a, b, c, d) along the column are significantly different (*P* ≤ 0.05), MRB—Maharashtra Rabi Bajra. FRAP—ferric reducing antioxidant power

### 3.6. Correlations of Yield and Antioxidant Components with Antioxidant Activity

The relationship of antioxidant activity with antioxidant components as well as the yield of methanolic extracts of raw and processed pearl millet is shown in Table 4. There was variation in the antioxidant activity analyzed by different assays and antioxidant components in K and MRB varieties. The results indicated significant negative correlations between methanolic extract (*r* = −0.629, *P* ≤ 0.01) as well as tannin content (*r* = −0.557, *P* ≤ 0.01) while, a positive relationship between flavonoid content (*r* = 0.712, *P* ≤ 0.01) and DPPH free radical scavenging activity existed. There was a strong positive relationship between reducing power and flavonoid content (*r* = 0.456, *P* ≤ 0.05) and FRAP with methanolic extracts (*r* = 0.478, *P* ≤ 0.05) suggesting that methanol was efficient in extracting bioactive components responsible for antioxidant activity. These results suggest that the DPPH radical scavenging activity and reducing power assay in the K variety was largely due to the presence of flavonoids. However, in MRB, antioxidant components such as phytic acid, tannin and flavonoids were poorly correlated with antioxidant activity as determined by three methods suggesting that these components did not contribute to the antioxidant activity of the MRB variety.

**Table 4:** Correlation of yield and antioxidant components with antioxidant activity of two pearl millet cultivars.

Pearl millet	Yield	Phytic acid	Tannins	Flavonoids
Kalukombu				
DPPH	−0.629 **	−0.130	−0.557 **	0.712 **
Reducing power	−0.361	0.222	−0.100	0.456 *
FRAP	0.478 *	−0.148	0.165	−0.078
Maharashtra Rabi Bajra				
DPPH	−0.317	−0.279	−0.121	−0. 282
Reducing power	−0.317	−0.273	−0.110	−0.282
FRAP	−0.322	0.276	−0.141	0.181

*—correlation is significant at the 0.05 level (2 tailed); **—correlation is significant at the 0.01 level (2 tailed), FRAP—Ferric reducing ant.

## 4. Conclusions

In conclusion, the antioxidant activity of pearl millet was influenced both by the processing methods and the cultivars. K and MRB showed low inhibition values of DPPH (about 60%), RPA (about 0.32) and FRAP (about 2.69). Between the cultivars, K exhibited a higher content of antioxidant components reflecting its higher antioxidant capacity. The Bran Rich Fraction showed high antioxidant activity in terms of RPA which was due to the tannin, phytic acid and flavonoid levels. The millet subjected to various heat treatments exhibited higher antioxidant activity (DPPH scavenging activity and RPA) mainly due to its flavonoid content. An inverse relationship was found between radical scavenging activity and yield. It was noteworthy that significantly (*P* ≤ 0.05) high radical scavenging activity in heat treated millet had the lowest yield, whilst germinated millet which showed the lowest activity had the highest yield.
